# Assessing the prognostic utility of smoldering multiple myeloma risk stratification scores applied serially post diagnosis

**DOI:** 10.1038/s41408-021-00569-2

**Published:** 2021-11-26

**Authors:** Alissa Visram, S. Vincent Rajkumar, Prashant Kapoor, Angela Dispenzieri, Martha Q. Lacy, Morie A. Gertz, Francis K. Buadi, Suzanne R. Hayman, David Dingli, Taxiarchis Kourelis, Wilson Gonsalves, Rahma Warsame, Eli Muchtar, Nelson Leung, Linda B. Baughn, Robert A. Kyle, Shaji Kumar

**Affiliations:** 1 Division of Hematology, Mayo Clinic Rochester, Mayo Clinic, MN USA; 2 Department of Medicine, Division of Hematology, The Ottawa Hospital, Ottawa Hospital Research Institute, Ottawa, ON Canada; 3 Division of Nephrology, Mayo Clinic Rochester, Mayo Clinic, MN USA; 4 Division of Laboratory Genetics and Genomics, Mayo Clinic Rochester, Mayo Clinic, MN USA; 5 Division of Laboratory Medicine and Pathology, Mayo Clinic, MN USA

**Keywords:** Epidemiology, Disease-free survival, Risk factors, Myeloma

## Abstract

The Mayo-2018 smoldering multiple myeloma (SMM) risk score is used routinely in the clinical setting but has only been validated at diagnosis. In SMM patients, the progression risk decreases over time. However, the utility of applying risk stratification models after diagnosis is unknown. We retrospectively studied 704 SMM patients and applied the Mayo 2018 and IMWG-2020 risk stratification models at annual landmark timepoints up to 5 years post diagnosis. The Mayo-2018 and IMWG-2020 models reliably stratified patients based on progression risk when applied post diagnosis. The respective 2-year progression risk in Mayo-2018 high risk patients versus IMWG-2020 intermediate-high risk patients was 51% versus 62% at the 1-year landmark and 47% versus 45% at the 4-year landmark. We showed that patients categorized at Mayo-2018 high-risk at follow-up had a similar risk of progression if the baseline risk assessment was low-intermediate versus high-risk (HR 1.04, 95% CI 0.46–2.36, *p* = 0.931 at 5-year landmark). Patients migrating to a higher risk category during follow up had a higher progression risk compared to patients with stable/decreased risk categorization. Our findings support the use of these risk scores post-diagnosis and suggest that patients evolving to a high-risk category may benefit from early intervention therapeutic approaches.

## Introduction

Smoldering multiple myeloma (SMM) is a precursor clinical disorder on the spectrum between monoclonal gammopathy of undetermined significance (MGUS] and multiple myeloma (MM). The definition of SMM has evolved with time. In 2014, in an effort to diagnose patients prior to developing end organ damage, the International Myeloma Working Group (IMWG) revised the MM diagnostic criteria to re-classify ultra-high risk SMM patients (those with an estimated 2-year risk of progression >80% to MM) as MM. The updated “SLiM” diagnostic criteria for MM include patients with a baseline bone marrow plasma cell burden (BM PC) ≥60%, free light chain ratio (FLCr) ≥100 with involved FLC ≥ 10 mg/dL, or >1 focal lesion on MRI [1]. SMM, however, is defined as the presence of a monoclonal protein (MCP) ≥3 g/dL and/or a bone marrow plasma cell (BM PC) burden between 10–59%, in the absence of myeloma defining events (hypercalcemia, anemia, renal failure, lytic bone disease, or the updated SLiM criteria outlined above) [1].

Until recently, the standard of care for management of SMM patients was active surveillance to monitor for myeloma defining events that would warrant therapy. However, the QUIREDEX and ECOG E3A06 phase 3 clinical trials demonstrated that patients with high-risk SMM had a longer time to progression to MM with early therapeutic intervention, and early intervention with lenalidomide had limited toxicity [2–4]. Given these findings, there are a multitude of ongoing trials investigating two early intervention strategies: lower intensity therapy to delay progression to active MM, and aggressive therapy to eradicate the plasma cell clone and prevent progression. While novel treatment regimens have more limited toxicities compared to conventional chemotherapy, in this otherwise asymptomatic patient population the financial implications, treatment-related toxicities and quality of life impacts of early intervention need to be carefully weighed against the benefits. Therefore, there is a need to optimize the use of SMM risk stratification models to accurately identify high-risk SMM patients that may benefit from treatment.

SMM risk stratification systems commonly incorporate surrogate measures of tumor burden, such as the MCP or BM PC quantification, to prognosticate the progression risk. The Mayo 2018 risk stratification model was used in the ECOG E3A06 trial and is commonly used in the clinical setting [5]. In a multinational effort to optimize prognostication, in 2020 the IMWG validated the Mayo 2018 score and also developed a novel risk stratification score incorporating a more refined classification of risk factors included in the Mayo 2018 model, as well as high risk cytogenetics [6]. A significant limitation of both the Mayo 2018 and IMWG 2020 SMM risk scores is that they were derived based on disease characteristics at SMM diagnosis, and therefore assume that progression risk remains constant over time. However, retrospective studies have shown that progression risk is highest within the first 5 years of SMM diagnosis, and then stabilizes at 3–5% annually thereafter [5, 7]. If the risk of progression to MM decreases with time, re-applying risk stratification scores post-diagnosis may not accurately stratify high risk SMM patients. Therefore, the aim of this study was to assess whether the Mayo 2018 and IMWG 2020 scores could be used dynamically to risk stratify patients post-diagnosis, and whether they could identify SMM patients with evolving disease.

## Methods

We used the prospectively maintained Mayo Clinic database to identify SMM patients diagnosed with smoldering multiple myeloma between 1 January 2000 and 10 January 2020. Electronic medical records were retrospectively reviewed to ensure that study patients met inclusion and exclusion criteria. Patients were included if the baseline MCP was ≥3 g/dL or BM PC burden was between 10 and 59%. SMM patients with a baseline FLCr ≥100 and involved FLC ≥10 mg/dL or baseline BM PC ≥ 60% were excluded, as they would have met the revised MM criteria [1]. Additionally, SMM patients treated with plasma-cell directed therapies within 3 months of diagnosis were excluded. Serial FLC, MCP, and BM PC data (when available) were collected for SMM patients. Given limitations in sample size, advanced imaging to exclude focal lesions was not a requirement for study inclusion.

The Mayo 2018 SMM risk score incorporates three risk factors: involved to uninvolved FLCr >20, MCP > 2 g/dL, and BM PC > 20% [5]. Categorization of SMM patients was as follows: low risk (0 risk factors), intermediate risk (1 risk factor), high risk (≥2 risk factors). The IMWG 2020 SMM risk score incorporates 4 risk factors: involved to uninvolved FLCr (0–10 is 0 points, >10–25 is 2 points, >25–40 is 3 points, and >40 is 5 points), MCP quantification (0–1.5 g/dL is 0 points, >1.5–3 g/dL is 3 points, and >3 g/dL is 4 points), BM PC (0–15% is 0 points, >15–20% is 2 points, >20–30% is 3 points, >30–40% is 5 points, and >40% is 6 points), and high risk fluorescence in situ hybridization (FISH) markers [presence of *t* (4;14), *t* (14;16), gain 1q, or del[13q]/monosomy 13 is 2 points]. Categorization of SMM patients using IMWG 2020 score was as follows: low risk (0–4 points), low-intermediate risk (5–8 points), intermediate risk (9–12 points), and high risk (>12 points) [6].

The Mayo 2018 and IMWG 2020 SMM risk scores were used to re-stage patients at 1, 2, 3, 4, and 5 years post diagnosis (±4 months). The Freelite test (Binding Site, Birmingham, United Kingdom) was used to quantify FLC, and the reference range for serum kappa light chains was 0.33–1.94 mg/dL, and 0.57–2.63 mg/dL for serum lambda light chains [8]. Given that repeat bone marrow biopsies are not the standard of care in SMM, annual bone marrow biopsies were not available for all patients. The most recent BM PC quantification was used when re-applying the SMM risk score during follow up. If repeat FISH data was not available within 4 months of each annual re-assessment, we made the following assumptions: Patients with a detectable primary cytogenetic abnormality (*t* (4;14) or *t* (14;16)) at any time during SMM follow up were assumed to be positive for the specific abnormality for the entire SMM disease course. If an IgH break apart probe (BAP) was normal and ≥50 plasma cells were analyzed prior to disease progression, we assumed that the sample was negative for *t* (4;14) and *t* (14;16) at diagnosis and follow up. After a secondary cytogenetic abnormality (gain 1q or del13q/monosomy 13) was detected, patients were assumed to be positive for that abnormality for the remainder of their SMM follow up. Patients with at least 1 detectable high risk cytogenetic marker [*t* (4;14), *t* (14;16), del13q, monosomy13, or gain1q] were considered evaluable for risk stratification using the IMWG 2020 score, irrespective of whether data was available for all high risk IMWG 2020 FISH markers. However, if no high-risk FISH abnormality was detected, only patients with cytogenetic results for IgH translocations (specifically *t* (4;14) and *t* (14;16) if an IgH BAP was abnormal), del13q/monosomy13, and gain1q were evaluated using the IMWG 2020 score.

Baseline characteristics were summarized using descriptive statistics. Time to event analyses were performed using the Kaplan–Meier method, and survival differences between groups were assessed using the Log rank test. Time to progression (TTP) was defined as time from diagnosis or landmark time point (1, 2, 3, or 4 years post diagnosis) to development of end organ damage attributed to myeloma (the CRAB features and cutoffs outlined in the IMWG 2014 criteria [1]), or treatment initiation for systemic AL amyloidosis or multiple myeloma in the absence of CRAB features. Patients were censored if treated for SMM on a clinical trial, or at last follow up. Follow-up time was based on the reverse Kaplan–Meier method [9]. Cox proportional hazards analyses were used to provide a hazard ratio of the TTP based on the SMM risk score at pre-specified timepoints. A parallel plot was used to visualize the fluctuations in SMM risk score assignment within individuals over time. Concordance between risk categorization was assessed using the Cohen’s kappa test for agreement. For all tests, a two-sided *p*-value < 0.05 was considered statistically significant. Statistical analysis was performed using JMP Pro version 14.1.0 (SAS, Cary, NC). This study was approved by the Mayo Clinic institutional review board.

## Results

A total of 704 SMM patients with available baseline sFLC, MCP, and bone marrow biopsy results were included in this study. Cross-sectional imaging (whole-body MRI, spine and pelvis MRI, PET scan, or whole-body low dose CT scan) confirming the absence of focal lytic lesions at diagnosis, or prior to progression was available for 496 (70%) patients. The remaining 208 (30%) had an unremarkable x-ray skeletal survey. Additional baseline characteristics of included patients are summarized in Table 1. Overall, 170 (24%) patients had at least 1 repeat bone marrow biopsy for plasma cell quantification during the first 5 years of SMM follow up. The median TTP was 6.4 (IQR 5.3–8.2) years for the entire cohort, and at the end of follow up, 316 patients were treated due to progression (19 for systemic light chain amyloidosis, 192 for symptomatic MM, 105 for SLiM criteria or rapidly evolving monoclonal proteins) whereas 388 were censored. Of the eight patients with IgM SMM, two met criteria for progression (one for symptomatic IgM MM, and one for systemic AL amyloidosis).

**Table 1:** Baseline SMM patient characteristics.

SMM patients (*n* = 704)	
Median age at diagnosis - *n* (IQR)	65.3 (57.3–71.9)
Sex	
Male -*n* (%)	418 (59.4)
Female - *n* (%)	286 (40.6)
Cross-sectional imaging available prior to progression^a^ - *n* (%)	496 (70.5)
Heavy chain isotype	
IgG - *n* (%)	538 (76.4)
IgA - *n* (%)	120 (17)
IgM - *n* (%)	8 (1.1)
Biclonal - *n* (%)	12 (1.7)
Light chain isotype	
Kappa - *n* (%)	461 (65.5)
Lambda - *n* (%)	243 (34.5)
Baseline diagnostics	
Median MCP - g/dL (IQR)	1.7 (1–2.5)
Median BM PC - % (IQR)	15 (12–22)
Median sFLCr - *n* (IQR)	8 (2.7–25.6)
Cytogenetics	
del[13q] or monosomy 13^b^	122 (32.2)
*t* (4;14)^b^	39 (10)
*t* (14;16)^b^	14 (3.6)
Gain[1q]^b^	61 (32.8)

^a^Advanced imaging was defined as whole-body low dose CT, PET-CT scan, or MRI (including at least the spine and pelvis).

^b^At baseline, cytogenetic data was available for 379 patients for monosomy 13 or deletion 13q, 390 patients for *t* (4;14), 385 patients for *t* (14;16), and 186 patients for gain [1q].

### Mayo 2018 SMM risk stratification

**Fig. 1:**
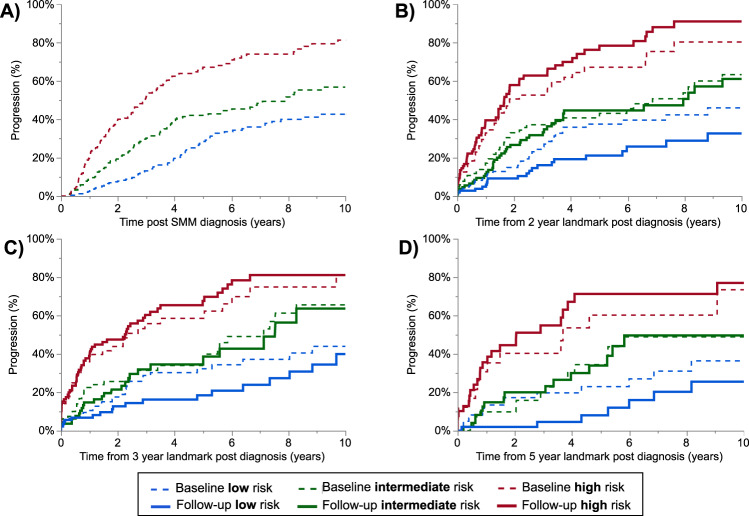
Time to progression (TTP) stratified by the Mayo 2018 risk score. SMM patients were stratified by the Mayo 2018 score based on the MCP, FLCr, and BMPC at diagnosis (dashed lines) or the updated values at the post-diagnosis timepoint (solid lines). The TTP is at baseline (**A**), and post-diagnosis landmarks of 2 years (**B**), 3 years (**C**), and 4 years (**D**). At each timepoint there was a significant difference in the TTP based on risk categorization is based on baseline or follow up risk factors (log rank *p* < 0.001).

The median follow-up prior to progression for the 704 SMM patients included in the Mayo 2018 risk stratification was 5.7 (95% CI 5.2–6.5) years. At diagnosis, 271 (38%) patients were low risk, 228 (32%) were intermediate risk, and 205 (29%) were high risk based on the Mayo 2018 risk score. The SMM risk score was re-assessed post-diagnosis in patients that had not progressed (430 patients 1 year post diagnosis, 326 patients at 2 years post diagnosis, 260 patients at 3 years post diagnosis, 203 patients at 4 years post diagnosis, and 147 patients at 5 years post diagnosis). We assessed the TTP from diagnosis and from landmark timepoints during follow-up, in Fig. 1. The TTP was stratified by the SMM categorization at diagnosis (the dashed lines) as well as the re-categorization based on lab/pathology values at each landmark timepoint during follow up (the solid lines). We showed that if only diagnostic values were used in risk categorization, the TTP between risk categories became less distinct over time. However, when follow-up values were used to re-stratify patients, the TTP between risk categories was more consistent over time (point estimates and progression risks are summarized in Table 2). When the SMM score was re-assessed during follow up, high risk SMM patients had a risk of progression approximately five times higher than low risk patients, and this risk remained relatively stable over time. The Mayo 2018 was able to consistently risk stratify patients over time even in the subset of patients with cross-sectional imaging demonstrating the absence of lytic lesions prior to progression, as shown in supplementary figure S1.

**Table 2:** Summary of risk of progression over time, grouped by the SMM risk categorization.

		Mayo 2018 low risk (score 0), IMWG 2020 low risk (score ≤4)				Mayo 2018 intermediate risk (score 1), IMWG 2020 low-intermediate risk (score 5–8)				Mayo 2018 high risk (score ≥2), IMWG 2020 intermediate & high risk (score ≥9)^b^			
Years post diagnosis	Total *n*	*n*	2-year prog (%)	5-year prog (%)	HR	*n*	2-year prog (%)	5-year prog (%)	HR^a^ (95% CI), *p* value	*n*	2-year prog (%)	5-year prog (%)	HR^a^ (95% CI), *p* value
Mayo 2018 score (5)													
Baseline	704	271	7.8	28.4	Ref	228	19.2	41.8	1.7 (1.2–2.2), *p* < 0.001	205	40	67.1	3.3 (2.5–4.4), *p* = <0.001
1 year	430	142	10	27.1	Ref	146	27.5	47.3	2.3 (1.5–3.5), *p* < 0.001	142	50.9	72.8	5.1 (3.5–7.6), *p* = <0.001
2 years	326	112	9.1	21.1	Ref	115	26.7	44.6	2.1 (1.3–3.5), *p* = 0.003	99	59.5	78.4	5.9 (3.6–9.6), *p* = <0.001
3 years	260	90	12.7	18.5	Ref	83	21.4	34.5	1.8 (1–3.1), *p* = 0.054	87	47.4	67.6	4.2 (2.6–6.9), *p* = <0.001
4 years	203	74	9.9	12.3	Ref	78	20.9	32	1.9 (1–3.8), *p* = 0.05	51	47.2	65.3	4.7 (2.5–9.1), *p* = <0.001
5 years	147	54	1.9	8	Ref	55	19.7	33.5	2.8 (1.2–6.4), *p* = 0.013	38	45.9	72.2	7.4 (3.4-15.8), *p* = <0.001
IMWG 2020 score (6)													
Baseline	264	90	9.1	32.5	Ref	111	21.4	49.3	2.3 (1.3–3.9), *p* = 0.003	63	49.9	79.5	5.1 (2.9–8.9), *p* < 0.001
1 year	197	58	14.4	36.1	Ref	68	22.3	49.3	1.2 (0.8–2.8), *p* = 0.179	71	62.4	80	4.3 (2.4–7.5), *p* < 0.001
2 years	143	44	12	23.9	Ref	56	28.6	44.9	2 (0.9–4.3), *p* = 0.095	43	64.5	89.3	5.4 (2.5–11.6), *p* < 0.001
3 years	106	32	16.4	22.3	Ref	36	19.3	54.2	1.7 (0.6–4.2), *p* = 0.292	38	62.2	71.7	4.5 (1.9–10.6), *p* < 0.001
4 years	73	23	18.6	30.2	Ref	26	27.3	56.4	1.8 (1–3.3), *p* = 0.043	24	45.1	45.1	2.3 (0.8–6.7), *p* = 0.115

^a^The comparator group is the low-risk SMM patients.

^b^Given the limited sample size of IMWG 2020 high-risk patients over time (*n* = 9 at baseline, *n* = 15 at the 1 year landmark, *n* = 9 at the 2-year landmark, *n* = 7 a the 3-year landmark, *n* = 8 at the 4-year landmark), the intermediate and high risk IMWG 2020 groups were combined.

**Fig. 2:**
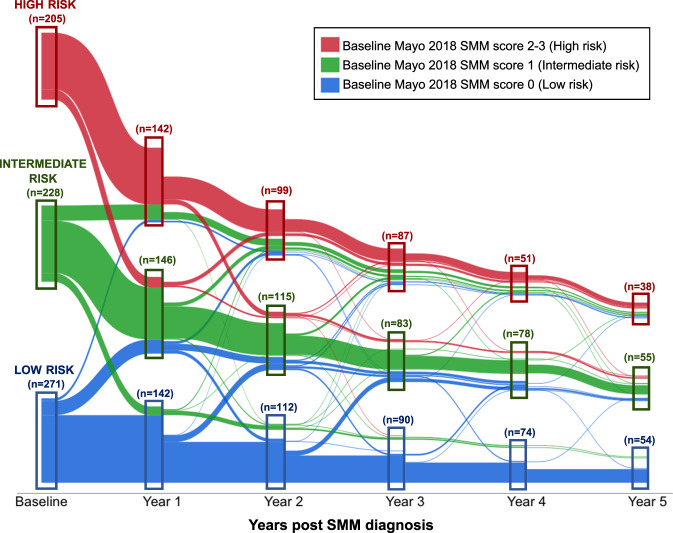
Parallel plot demonstrating the change in the Mayo 2018 SMM risk score over time. The colored lines represent patients based on their baseline SMM risk stratification, as shown in the legend. The line thickness is proportional to the number of patients within each SMM score stratum. At each annual time point post SMM diagnosis, the boxes represent the composition of patients re-categorized at high risk (red box), intermediate risk (green box), or low risk (blue box). This plot demonstrates that in some SMM patients, the risk categorization is dynamic over time.

**Fig. 3:**
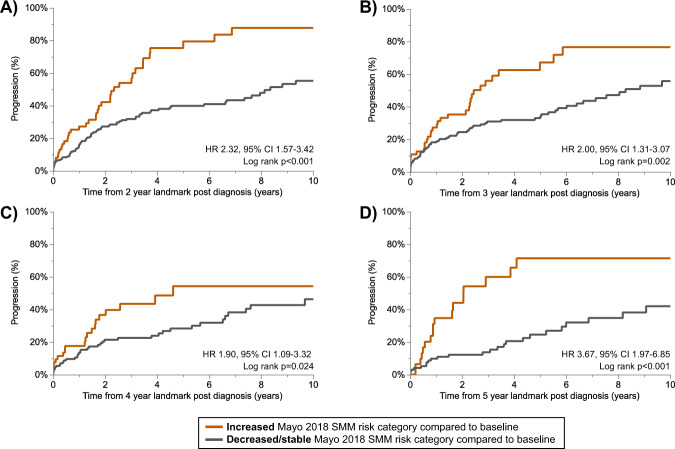
The time to progression, stratified by migration of SMM Mayo 2018 risk category during follow up. Patients were grouped based on whether the Mayo 2018 category at follow up was increased or stable/decreased compared to baseline. The stage migration of SMM patients without progression at 2 years (**A**), 3 years (**B**), 4 years (**C**), and 5 years (**D**) post SMM diagnosis is shown. The percentage of patients evolving to a higher risk category was 20% at the 2-year landmark, 23% at the 3-year landmark, 29% at the 4-year landmark, and 24% at the 5-year landmark.

To assess whether risk categorization remained stable within patients over time, we created a parallel plot (shown in Fig. 2) to visually summarize the SMM risk score of patients when re-evaluated during annual follow up. This figure demonstrated that the SMM risk categorization could change over time in some patients. For example, when the SMM was re-assessed at 1 year after SMM diagnosis, 142 patients were classified as high risk; however, at SMM diagnosis 4 (3%) of these patients had a low risk score, and 28 (20%) had an intermediate risk score. Importantly, high risk patients seldom became low risk during follow up. To assess the prognostic significance of an increase in SMM risk category, we stratified patients based on whether the SMM risk category at follow-up had increased versus was stable or decreased compared to the baseline SMM risk stratification. As shown in Fig. 3, patients evolving to a higher SMM risk score during follow-up consistently had an increased risk of progression to MM or amyloidosis. At each follow-up timepoint, ~20–30% of SMM patients had evolved to a higher risk category. When restricting to patients with a low or intermediate risk categorization at SMM diagnosis, we found that the risk of progression was approximately three times higher in patients evolving to the high-risk category during follow up compared to if patients remained low or intermediate risk (HR 3.34, 95% CI 2.04–5.48, *p* < 0.001 at 2-year landmark; HR 3.41, 95% CI 2.05–5.68, *p* < 0.001 at 3-year landmark; HR 2.69, 95% CI 1.34–5.38, *p* = 0.005 at 4-year landmark; and HR 4.73, 95% CI 2.26–9.88, *p* < 0.001 at 5-year landmark). Conversely, SMM patients categorized as high-risk during follow-up had a similar risk of progression regardless of their baseline risk stratification (high-risk vs low/intermediate risk); HR 0.87, 95% CI 0.51–1.48, *p* = 0.606 at 2-year landmark; HR 1.01, 95% CI 0.59–1.73, *p* = 0.972 at 3-year landmark; HR 0.75, 95% CI 0.34–1.65, *p* = 0.467 at 4-year landmark; and HR 1.04, 95% CI 0.46–2.36, *p* = 0.931 at 5-year landmark.

### IMWG 2020 SMM risk stratification

At baseline 189 patients had complete data to evaluate for all cytogenetic markers included in the IMWG 2020 risk stratification [del[13q]/monosomy 13, *t* (4;14), *t* (14;16), and gain1q], and 87 (46%) had at least 1 high risk abnormality. However, after including patients with a high-risk FISH abnormality at baseline (even if not all cytogenetic abnormalities were assessed), 264 patients were evaluable for IMWG 2020 risk stratification at baseline. At diagnosis, 90 (34%) of patients were low risk, 111 (42%) were low-intermediate risk, 54 (21%) were intermediate risk, and 9 (3%) were high risk. All nine patients categorized as high risk by the IMWG 2020 score were also high risk using the Mayo 2018 scoring. In the initial IMWG 2020 publication, the 2-year risk of progression for SMM patients with an IMWG 2020 intermediate risk score or higher was ~45%, which was roughly equivalent to the 2-year risk of progression for Mayo 2018 patients. Therefore, we dichotomized the two risk score categories (IMWG 2020 score <9 vs ≥9, and Mayo 2018 score <2 versus ≥2) and found that the 2 staging systems were moderately concordant (kappa coefficient 0.61, 95% CI 0.51–0.72, *p* < 0.001).

**Fig. 4:**
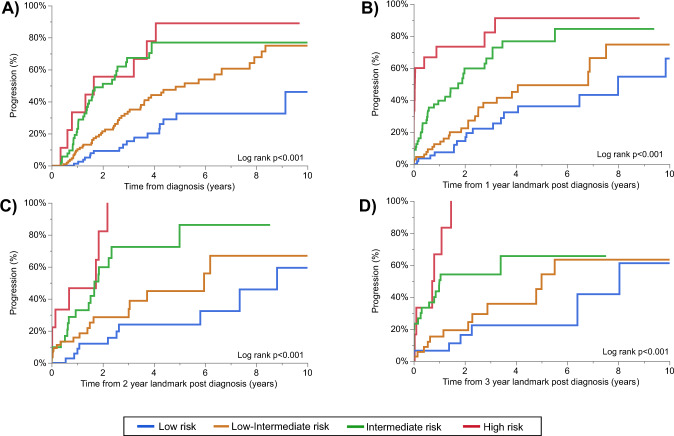
The TTP over time based on the IMWG 2020 risk stratification. The TTP is presented stratified by the SMM risk score at baseline (**A**), and post-diagnosis landmarks of 1 year (**B**), 2 years (**C**), and 3 years (**D**). The risk score was re-assessed based on updated data at each time point.

**Fig. 5:**
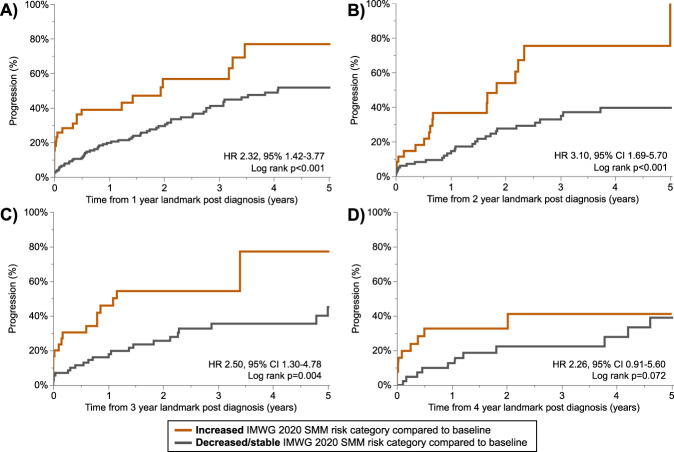
The time to progression, stratified by migration of SMM IMWG 2020 risk category during follow up. Patients were grouped based on whether the IMWG 2020 category at follow up was increased or stable/decreased compared to baseline. The stage migration of SMM patients without progression at 1 year (**A**), 2 years (**B**), 3 years (**C**), and 4 years (**D**) post SMM diagnosis is shown. The percentage of patients evolving to a higher risk category was 20% at the 1-year landmark, 26% at the 2-year landmark, 30% at the 3-year landmark, and 36% at the 5-year landmark.

The median follow-up from SMM diagnosis for patients evaluable for risk stratification using the IMWG 2020 score at baseline was 4.1 (95% CI 3.4–4.7) years. Due to the limited follow up and sample size of patients evaluable for the IMWG 2020 risk score, this score was only re-assessed for 4 years post diagnosis. As shown in Fig. 4, when re-applied post diagnosis the IMWG 2020 risk score was able to consistently stratify patients with an intermediate and high-risk patients (those with a 2y TTP of ≥50%) from low and low-intermediate risk patients (those with a 2-year TTP of <50%). The 2-year and 5-year TTP remained relatively similar over time, as shown in Table 2. Compared to low-risk patients, intermediate-high risk patients had a 4–5 fold increase in the risk of progression when the IMWG score was applied up to 3 years post SMM diagnosis. To assess whether changes in risk categorization had implications on prognosis, we stratified patients into 2 groups; those who migrated to a higher risk category at follow up compared to baseline, and those whose risk categorization was either stable or was lower than at baseline. We found that evolved to a higher risk category at follow up assessment tended to have an increased risk of progression compared to patients whose risk category was stable or decreased compared to baseline (shown in Fig. 5**:** HR 2.32, 95% CI 1.42–3.77, *p* < 0.001 at 1-year landmark; HR 3.10, 95% CI 1.69–5.70, *p* < 0.001 at 2-year landmark; HR 2.50, 95% CI 1.30–4.78, *p* = 0.004 at 3-year landmark; and HR 2.26, 95% CI 0.91–5.60, *p* = 0.072 at 4-year landmark).

## Discussion

This is the largest study to evaluate risk stratification in SMM beyond diagnosis. In this study we showed that both the Mayo 2018 and IMWG 2020 risk scores reproducibly stratified patients based on the risk of progression, even when applied up to 5 years after diagnosis. We demonstrated that in some SMM patients the risk categorization is dynamic over time, and that patients with an “evolving” clinical presentation – those with an increasing risk categorization over time - are at a higher risk of progression. Expert consensus is that SMM patients with a 2-year risk of progression >50% may benefit from early intervention strategies. Therefore, it is important that we showed that both risk scores were able to reliably identify a risk group with a ~50% 2-year progression risk when re-evaluated during follow up. Furthermore, we show that in patients evolving to the Mayo 2018 high risk category, the risk of progression was similar regardless of the baseline risk assessment. This suggests that patients evolving to a high-risk category may also benefit from early intervention treatment strategies, and that SMM clinical trial eligibility may be broadened to include these patients. Therefore, we recommend that in patients with SMM, the risk stratification models should be re-applied during follow up, and treatment decisions should be made accordingly.

SMM is a clinically heterogeneous entity comprised of patients with a MGUS-like indolent phenotype and others destined to progress to MM. Though commonly used risk stratification models, such as the Mayo 2018 or PETHEMA models [5, 10], are applied at diagnosis, differentiating patients with an indolent versus more aggressive clinical course requires dynamic assessments. The idea of using serial assessments to identify patients with evolving biomarkers was first described by Rosiñol et al. in 2003 [11]. Since then, multiple groups have aimed to define risk stratification systems that incorporate serial markers of clonal burden to identify patients at highest risk of MM progression [12–16]. In 2016, Ravi et al., published that SMM patients with both an evolving MCP (≥10% and >0.5 g/dL increase in MCP within 6 months of diagnosis if baseline MCP ≥3 g/dL, or ≥25%, and >0.5 g/dL increase in MCP within 12 months of diagnosis if baseline MCP < 3 g/dL) and evolving hemoglobin (≥0.5 g/dL decrease within 12 months of diagnosis) had a 2-year progression risk of 82% [12]. However, when externally validated in two independent datasets, the evolving hemoglobin definition was not identified as a risk factor for progression [13, 15], and the presence of evolving MCP and hemoglobin resulted in a 2-year progression risk of only 18.5% [13]. In 2018, Fernández de Larrea et al. showed that SMM patients with an evolving MCP (defined as a ≥10% increase in MCP within 12 month of diagnosis if baseline MCP ≥3 g/dL or within 3 years if baseline MCP <3 g/dL) had a risk of progression to symptomatic MM 5 times higher than patients without an evolving MCP (2-year progression risk 66% vs. 12%, respectively) [16]. In 2018 Wu *et al*. developed the Sinai SMM risk model which assessed progression risk based on the trajectory of biomarkers over time [14]. While this model may better represent the biological evolution of tumor markers, the lack of clear cutoffs to define high versus low-risk biomarkers makes it difficult to implement in clinical practice. Both the Fernández de Larrea and Wu et al. studies defined SMM using the 2003 IMWG definition, and therefore may have incorporated patients with SLiM criteria who would now be treated for MM [1, 17]. Furthermore, neither study has been externally validated which limits applicability of these models. Currently, there is a need for a prognostic model that can dynamically evaluate progression risk beyond diagnosis.

Prior studies have shown that the time to progression in SMM patients with a similar genomic landscape to MM is shorter [18–20]. These patients are truly “asymptomatic” MM and develop manifestations of MM as the tumor burden increases. Primary cytogenetic abnormalities such as IgH translocations are found in early plasma cell clones, and remain stable during progression [19]. However, acquisition of DNA repair pathway, MYC, and mitogen activated protein kinase pathway abnormalities may are associated with increased progression risk [18]. Combining genomic markers of progression, dynamic tumor burden assessments, and markers of the tumor microenvironment will be key to optimizing future SMM prognostication tools. However, this comprehensive risk stratification approach will be costly and will require validation. The Mayo 2018 risk stratification model has been externally validated and can easily be used in the clinical setting. Our study provides the first evidence that the Mayo 2018 and IMWG 2020 risk models can robustly stratify patients based on the risk of progression over time [5, 6].

The main limitation of our study is the lack of serial bone marrow assessments during follow up. The BM PC is a variable in both the Mayo 2018 and IMWG 2020 scoring systems, and so in the absence of updated BM PC data we assumed a constant PC burden and cytogenetic risk over time. This assumption likely led to systematic underestimation of the risk score, which may have amplified differences between high versus lower risk groups (as we would expect some high-risk patients to have been inaccurately classified as low-intermediate risk because of unidentified secondary cytogenetic abnormalities or increases in BM PC burden). However, annual bone marrow biopsies are invasive and not the standard of care in SMM monitoring, and so this assumption reflects clinical practice. Furthermore, we showed that despite limited follow up BM PC values, re-evaluation of the Mayo 2018 risk score with follow up biomarker data led to consistent risk stratification over time. Our evaluation of the IMWG 2020 score was limited due to insufficient samples for FISH analysis which reduced sample size, and the more recent assessment of gain1q led to a shorter clinical follow up. While our inclusion criteria did not require patients to have had cross-sectional imaging to confirm the absence of lytic lesions, and therefore we may have included patients with SLiM MM, we evaluated the Mayo 2018 scoring over time in the subset of patients with available imaging and found similar results.

In conclusion, we showed that the Mayo 2018 and IMWG 2020 risk stratification models can be applied for up to 5 years post SMM diagnosis. Furthermore, we showed that patients migrating to a higher risk category have an increased risk of progression, suggesting that if patients evolve to a high-risk score during follow-up, they should be considered for an early intervention treatment approach.

## Supplementary information

Supplementary figure 1
