# Supplementary material for: Assessing the prognostic utility of smoldering multiple myeloma risk stratification scores applied serially post diagnosis
Source: Blood Cancer J. 2021 Nov 26;11(11):186. doi: 10.1038/s41408-021-00569-2 (PMC8626440; doi:10.1038/s41408-021-00569-2)
Supplement: Supplementary file 1 — Supplementary figure 1 [file 41408_2021_569_MOESM1_ESM.pptx]

## Slide 1
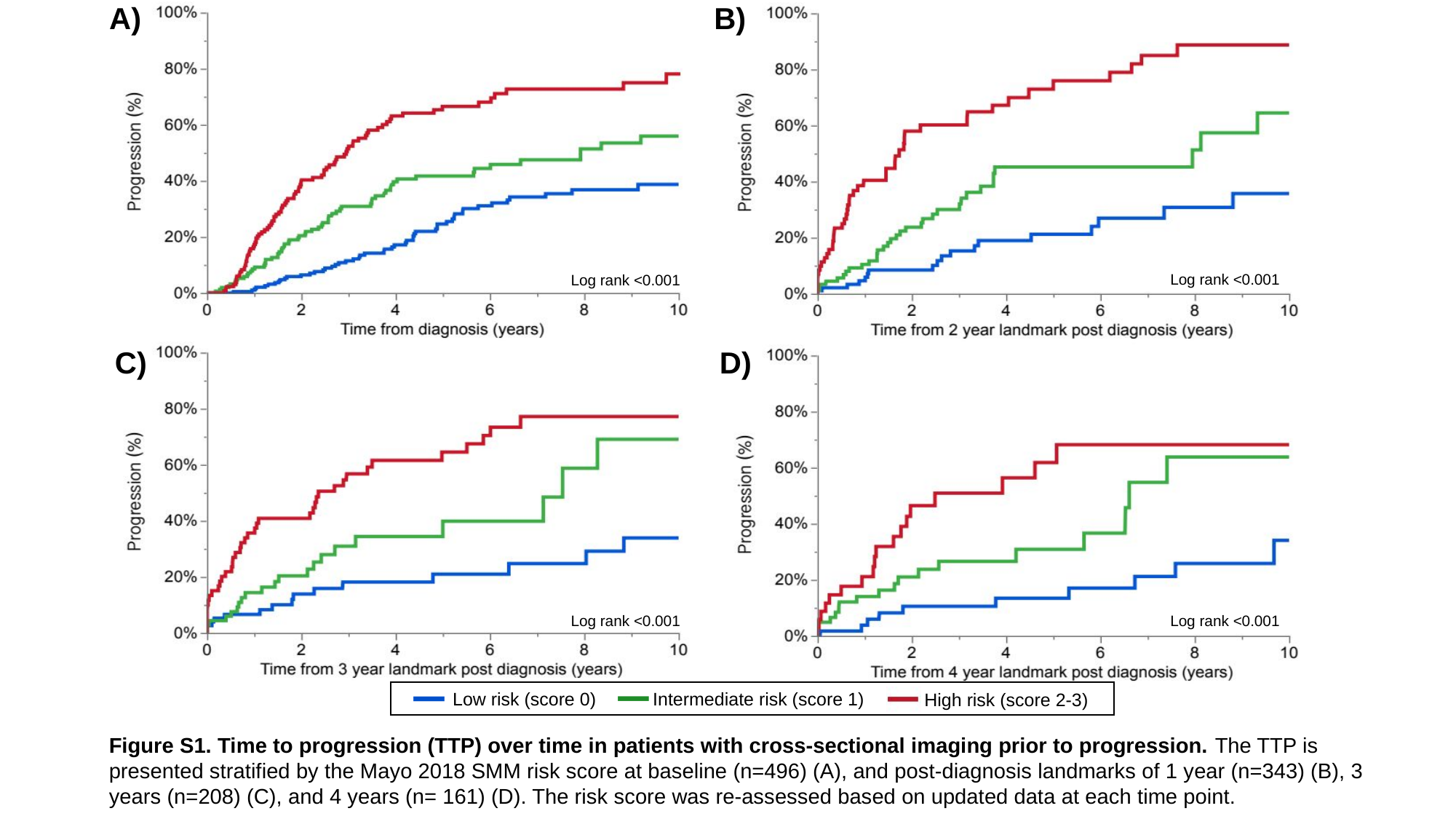

A)
B)
Log rank <0.001
Log rank <0.001
C)
D)
Log rank <0.001
Log rank <0.001
Low risk (score 0)
Intermediate risk (score 1)
High risk (score 2-3)
Figure S1. Time to progression (TTP) over time in patients with cross-sectional imaging prior to progression. The TTP is presented stratified by the Mayo 2018 SMM risk score at baseline (n=496) (A), and post-diagnosis landmarks of 1 year (n=343) (B), 3 years (n=208) (C), and 4 years (n= 161) (D). The risk score was re-assessed based on updated data at each time point.
